# All-cause and In-hospital Mortality after Aspirin Use in Patients Hospitalized with COVID-19: A Systematic Review and Meta-analysis

**DOI:** 10.1093/biomethods/bpac027

**Published:** 2022-10-29

**Authors:** Nischit Baral, Joshua D Mitchell, Pramod K Savarapu, Maxwell Akanbi, Bandana Acharya, Soumya Kambalapalli, Amith Seri, Krishna P Bashyal, Arvind Kunadi, Niranjan Ojha, Annabelle Santos Volgman, Tripti Gupta, Timir K Paul

**Affiliations:** Department of Internal Medicine, McLaren Flint/Michigan State University, Flint, MI, USA; Cardiovascular Division, Department of Medicine, Washington University in St. Louis, St. Louis, MO, USA; Department of Medicine, Ochsner Louisiana State University Health Shreveport-Monroe Medical Center, Monroe, LA, USA; Department of Internal Medicine, McLaren Flint/Michigan State University, Flint, MI, USA; Department of Health Sciences, Western New England University, Springfield, MA, USA; Department of Internal Medicine, McLaren Flint/Michigan State University, Flint, MI, USA; Department of Internal Medicine, McLaren Flint/Michigan State University, Flint, MI, USA; Department of Internal Medicine, McLaren Flint/Michigan State University, Flint, MI, USA; Department of Internal Medicine, McLaren Flint/Michigan State University, Flint, MI, USA; Department of Internal Medicine, Division of Cardiology, SUNY Upstate Medical University, NY, USA; Division of Cardiology, Department of Internal Medicine, Rush University Medical Center, Chicago, IL, USA; Department of Internal Medicine, Division of Cardiology, Vanderbilt University Medical Center, Nashville, TN, USA; Department of Cardiovascular Sciences, University of Tennessee College of Medicine, Nashville, TN, USA

**Keywords:** Aspirin, All-cause Mortality, In-hospital Mortality, COVID-19, Meta-analysis

## Abstract

**Background:**

With the results of the largest randomized controlled trial (RECOVERY) and the most extensive retrospective cohort study on COVID-19 recently published, we performed a meta-analysis on the association of aspirin with mortality of COVID-19. We aimed to investigate the role of aspirin in COVID-19 hospitalizations.

**Materials and Methods:**

We searched PubMed, EMBASE, and Cochrane databases for studies from January 1, 2020, until July 20, 2022, that compared aspirin versus non-aspirin use in hospitalized COVID-19 patients. We excluded case reports, review articles, and studies on non-hospitalized COVID-19 infections. We used the inverse variance method and random effects model to pool the individual studies.

**Results:**

Ten observational studies and one randomized controlled trial met the criteria for inclusion. There were 136,695 total patients, of which 27,168 were in the aspirin group, and 109,527 were in the non-aspirin group. Aspirin use was associated with a 14% decrease in all-cause mortality compared to non-aspirin use in patients hospitalized with COVID-19 (RR 0.86, 95% CI: 0.76-0.97, P = 0.002, I^2^= 64%). Among subgroups of studies reporting in-hospital mortality in COVID-19 hospitalizations, aspirin use was associated with a 16% decrease in in-hospital mortality compared to non-aspirin use (RR 0.84, 95% CI: 0.71-0.99, P = 0.007, I^2^= 64%).

**Conclusion:**

Our study shows that aspirin decreases in-hospital mortality in patients hospitalized with COVID-19. Further studies are needed to assess which COVID-19 patient populations benefit most, such as patients on aspirin for primary vs. secondary prevention of atherosclerotic disease. In addition, significant bleeding also needs to be considered when assessing the risk-benefit of aspirin use.

## 1. Introduction

The novel coronavirus disease 2019 (COVID-19) since first reported in November 2019 has emerged into a pandemic and resulted in more than one million deaths in the United States alone as of July 20, 2022 (1). Although most patients with COVID-19 have mild symptoms, the mortality rate for hospitalized patients remains high (1-5). Since the emergence of COVID-19, systemic corticosteroids for 7 to 10 days in patients with severe and critical COVID-19 (requiring mechanical ventilation or oxygen support), with a conditional recommendation not to use corticosteroid therapy in patients with non-severe COVID-19 (not requiring respiratory support or oxygen) was provided by WHO in September 2020 (1, 6).

Due to severe inflammatory response and hypercoagulability, the risk of thromboembolic events in COVID-19 are reported to be higher when compared to other acute medical illness or viral respiratory infections and is associated with a worse prognosis (7). Immune dysregulation with systemic inflammation (especially interleukin-6 along with complement activation) and thrombosis has been proposed for the pathogenesis of severe COVID-19 (8, 9). With platelet activation and sequestration in critical illnesses, the benefits of antiplatelet therapy secondary to the inhibition of platelet activation and accumulation have been studied extensively. Aspirin due to its antiplatelet and antiviral effects has demonstrated a reduction in replication, propagation, and infectivity of many RNA viruses such as MERS-CoV and CoV-229 E in both in-vitro and experimental models (4, 9-12).

Although aspirin was not among the guideline-recommended treatment for COVID-19 several observational studies along with one large randomized controlled trial (RCT) RECOVERY studied the beneficial effects of aspirin use on mortality in hospitalized patients with COVID-19 (3, 4, 11-15). The first PILOT study, the Collaborative Registry to Understand the Sequelae of Harm in COVID-19 (CRUSH-COVID) revealed that a combined exposure of pre-hospital and in-hospital aspirin use within the first 24 hours of admission led to a decrease in in-hospital mortality (4). In the Randomized Evaluation of COVID-19 Therapy in RECOVERY trial, aspirin was not found to be associated with a reduction in 28-day mortality in patients hospitalized with COVID-19. Still, there was a small increase in the rate of being discharged alive within 28 days (3). Following the publication of the RECOVERY trial (3) and a large observational cohort study (11), we conducted a meta-analysis to assess further the association between aspirin and all-cause mortality with the subgroup of in-hospital mortality in hospitalized patients with COVID-19.

## 2. Methods

We included RCTs, quasi-experimental, and retrospective cohort studies that reported hazard ratio, odds ratio, or relative risk of the effect of aspirin on all-cause mortality in patients hospitalized with COVID-19. We independently screened the manuscripts/full papers, abstracts, or titles of the studies from the electronic search to identify all potentially eligible studies and extracted data from PubMed/MEDLINE, Web of Science, Embase, and Google Scholar from January 1, 2020, until July 20, 2022, that fulfilled the eligibility criteria with no language restrictions, using the search terms ("aspirin" or "acetylsalicylic acid") and ("COVID 19" or "Novel Corona Virus Disease 2019" or "SARS COVID-19 Infection" or "Coronavirus Disease 2019 Virus" or "SARS-CoV-2 Infection"). The results from the Google scholar is combined with the results of the PubMed/MEDLINE because all the Google scholars articles were accessed using Pubmed.gov.

Eligible studies compared the use of aspirin versus no aspirin in patients hospitalized with COVID-19 and reported all-cause mortality. We excluded case reports, case series, review articles, and studies on non-hospitalized COVID-19 infections. The Preferred Reporting Items for Systematic Reviews and Meta-Analyses (PRISMA) statement for reporting systematic reviews as recommended by the Cochrane Collaboration was followed (16). Search results were saved in EndNote version X9 (Developer: Clarivate analysis) files. We extracted the data manually through a full-text review. Two reviewers (NB and PS) independently performed the title, abstract, and full-text screening. Conflicts were resolved through consensus; if not, the third author (MA) resolved the dispute.

We used the Newcastle-Ottawa Scale to assess the quality of observational studies (17). This scale assigns a maximum of nine points. We scored four for selecting and evaluating exposure, two for comparability, and three for assessing the outcome. If a study receives a score of six or higher, it is considered a high-quality publication with a low risk of bias (17). The RCT was assessed for quality using the Cochrane risk of bias tool (18). This tool covers six domains of bias: selection bias, performance bias, detection bias, attrition bias, reporting bias, and other biases. For the individual biases in the tool, the assessment of each bias is based on two parts– Support for Judgement and Review of authors' judgement. The Support for judgement provides a summary of characteristics of the trial based on which risk of each bias is determined and thus the transparency of the judgement is maintained. The second part of the tool involves assigning a judgment of high, low, or unclear risk of material bias for each item (18). The outcome of interest was all-cause mortality. We used the hazard ratio (HR) or odds ratio (OR), or relative risk (RR), depending on the studies, for the effect measure to generate the pooled risk ratio. We used adjusted HR or OR whenever they were reported. As done in previous studies, we directly incorporated HR as RRs while creating the forest plot (19-21). We have used the formula RR = OR/ {(1 – P0) + (P0 x OR)}, to transform ORs into RRs. In the formula, P0 is the incidence of the outcome of interest in the non-exposed group (19-21). To further, calculate the upper and lower confidence interval, we used the formula Standard of Error logarithmic (SElog) (RR) = SElog (OR) x log (RR)/ log (OR) (19-21). The analyses were performed using Review Manager 5.4 statistical software (Cochrane Collaboration, Oxford, U.K.) with an inverse variance method. We assessed the pooled RR and 95% confidence interval using the random effect model. In studies like systematic review and metanalysis, a treatment effect across various studies is investigated, the effects of treatments will not be the same across all populations (22). This variation in the effectiveness of treatments is referred to as treatment effect heterogeneity. The I-squared statistic is used as a measure to assess the amount of treatment effect heterogeneity (22). Sensitivity analysis was performed with the exclusion of individual studies to look for changes in the outcome. We used a statistical significance threshold of a P value of less than 0.05.

## 3. RESULTS:

### 3.1 Study selection:

Figure 1 shows the PRISMA flow diagram of study selection and inclusion.

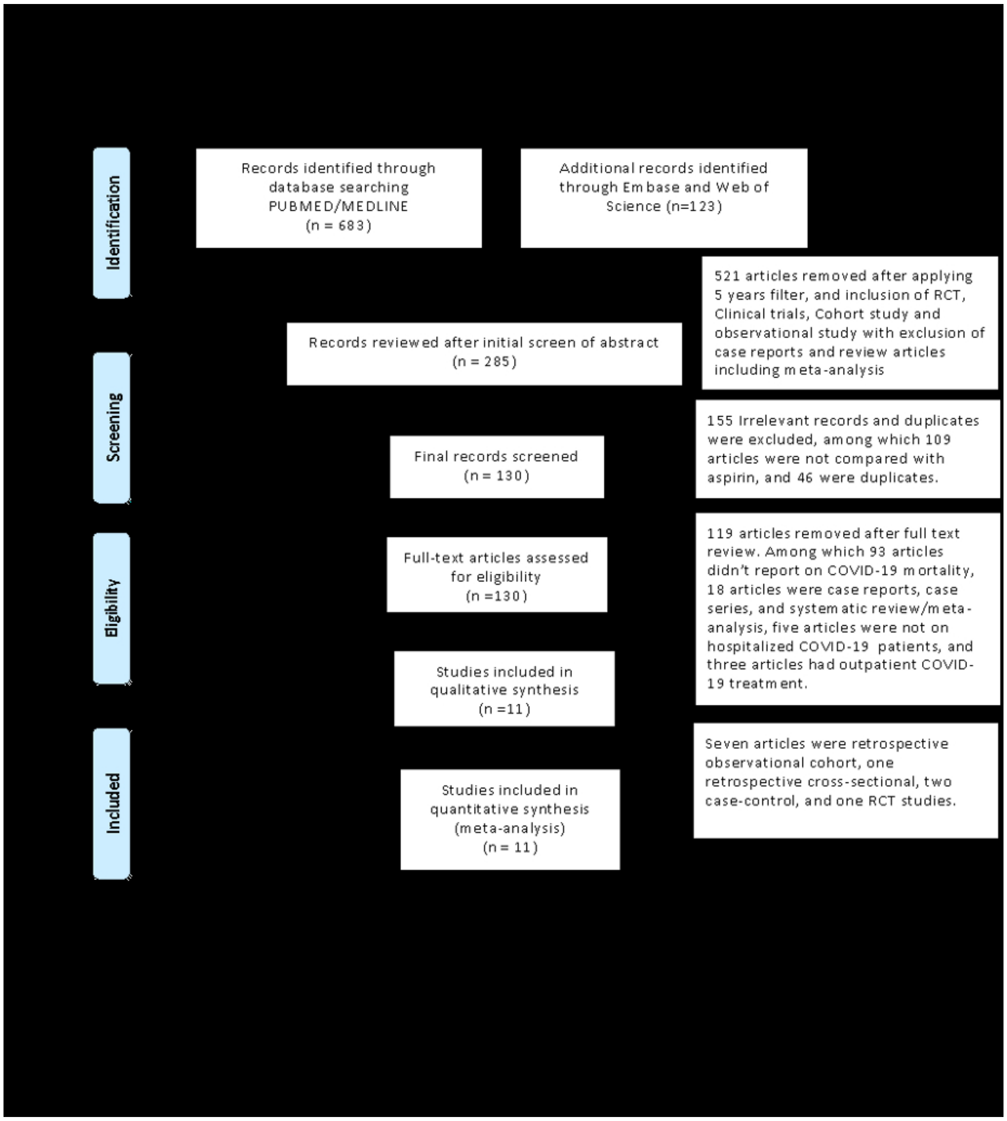

Figure 1: The PRISMA flow diagram of the studies in the meta-analysis; RCT - randomized controlled trial.

All studies reported all-cause mortality as in-hospital, in-hospital 28-day mortality, 30-day mortality, 60-day mortality, or overall mortality. The included studies' baseline characteristics are listed in Table 1.

| First author and year | Country | Design | No of participants (Aspirin/Non-Aspirin) | Aspirin dose (mg) | Mean age (years) | Study quality | Outcome | Covariates adjusted |
|---|---|---|---|---|---|---|---|---|
| Chow et al. 2020(4) | USA | Retrospective cohort study | 98/314 | 81 | 55 | 8 | In-hospital Mortality | Age, sex, BMI, race, comorbidities, home beta-blocker use |
| Yuan et al. 2020(14) | China | Retrospective | 52/131 | 150 | 71.2 | 7 | In-hospital Mortality | Age, sex. comorbidities |
| Liu et al. 2021(12) | China | Case-control | 24/24 | 100 | 54 | 8 | 30- and 60-day mortality | Propensity score matched on Age, gender, comorbidities |
| Meizlish et al. 2021(13) | USA | Retrospective | 319/319 | 81 | 70 | 8 | In-hospital Mortality | Propensity score matched on age, sex, obesity, anticoagulation, ICU stay, race and cardiovascular disease. |
| Chow et al. 2022(11) | USA | Retrospective | 15272/96997 | 81 | 63 | 8 | In-hospital 28-day mortality | Propensity score balanced on age, sex, race, comorbidities and history of aspirin use in previous 90 days |

| | | | | | | | | |
|---|---|---|---|---|---|---|---|---|
| RECOVERY 2021(3) | USA | RCT | 7351/7541 | 150 | 59.2 | High quality | 28-day mortality | Not applicable in RCT |
| Vahedian-Azimi et al. 2021(23) | Iran | Cohort Study | 337/250 | NA | 54.9 | 7 | In-hospital mortality | Age, sex, lockdown, drugs |
| Formiga et al. 2021(24) | Spain | Cohort study | 3291/2885 | NA | 68.5 | 7 | In-hospital mortality | Age, sex, comorbidities |
| Aghajani et al. 2021(25) | Iran | Cohort study | 366/655 | 80 | 61.6 | 8 | In-hospital mortality | Age, sex, comorbidities |
| Alamdari et al. 2020(26) | Iran | Retrospective cross-sectional | 53/406 | NA | 61.8 | 6 | In-hospital mortality | Not adjusted for covariates |
| Viecca et al. 2020(27) | Italy | Case-control | 5/5 | 1st 250, then 75 | 61.8 | 6 | 30-day mortality | Not adjusted for covariates |

**Table 1. Baseline characteristics of included studies**

**Abbreviations: BMI**– body mass index, **ICU–** intensive care unit, **RCT**- randomized controlled trial

Ten observational studies, and one RCT met the criteria for inclusion. There were 136,695 total patients, of which 27,168 were in the aspirin group and 109,527 were in the non-aspirin group. Aspirin use was associated with a 14% decrease in all-cause mortality compared to non-aspirin use in patients hospitalized with COVID-19 (RR 0.86, 95% CI: 0.76-0.97, P=0.002, $I^2$= 64%) (Figure 2).

**Figure 2: Forest plot of the effect of aspirin use on overall and in-hospital mortality in adults hospitalized with COVID-19.**

**CI: Confidence Interval SE: Standard Error**

Among subgroups of studies reporting in-hospital mortality in COVID-19 hospitalizations, aspirin use was associated with a 16% decrease in in-hospital mortality compared to non-aspirin use (RR 0.84, 95% CI: 0.71-0.99, P=0.007, $I^2$= 64%). However, aspirin was not associated with a statistically significant decrease in mortality compared to non-aspirin in a subgroup of studies that included out-of-hospital mortality after hospitalization for COVID-19 (RR 0.56, 95% CI: 0.22-1.41, P=0.05, $I^2$= 67%).

Sensitivity analysis:

The sensitivity analysis showed no change in statistical significance of odds ratios on aspirin's role in preventing all-cause mortality in COVID-19 hospitalized patients with the exclusion of any individual studies. However with the combined exclusion of Chow et al. (2020)(4), Aghajani et al.(25), and Meizlish et al.(13) studies, the odds ratio was statistically non-significant. However, there was no change in the overall beneficial role of aspirin with the exclusion of other studies. We used the exclusion method of individual studies and calculated the pooled RR.

Publication bias and Heterogeneity:

The funnel plot in our meta-analysis shows asymmetry in the distribution of the included studies due to the high heterogeneity of the included studies. The blue dotted line represents the odds ratio with 95% confidence interval. (Figure 3).

**Figure 3: Funnel plot for the assessment of publication bias of the included studies. The blue dotted line represents the odds ratio of all-cause mortality. SE: Standard of the Error log: logarithmic RR: Relative Risk**

Quality of included studies:

The quality of included observational studies is shown in Table 2, and the quality of included RCT is shown in Figure 4.

| Included Studies | Selection | | | | Comparability | | Outcome | | Classification |
|---|---|---|---|---|---|---|---|---|---|
| **Study (Year)** | **Representativeness of exposure group** | **Representativeness of non-exposed group** | **Ascertainment of exposure** | **Determination that outcome not present initially** | **Comparison of cohorts** | **Assessment of outcome** | **Long enough follow up** | **Adequacy of follow up?** | **Points** |
| Chow (2020) | Yes | Yes | Secure-Record | Yes | Yes | Reported | Yes | Yes | 8 |
| Yuan (2020) | Yes | Yes | Secure-record | Yes | Yes | Reported | No | Yes | 7 |

| | | | | | | | | | |
|---|---|---|---|---|---|---|---|---|---|
| Liu (2021) | Yes | Yes | Secure-record | Yes | Yes | Reported | Yes | Yes | 8 |
| Meizlish (2021) | Yes | Yes | Secure-record | Yes | Yes | Reported | Yes | Yes | 8 |
| Alamdari (2020) | Yes | Yes | Secure-Record | Yes | Yes | Reported | No (NA) | NA | 6 |
| Viecca (2020) | Yes | Yes | Secure-Record | Yes | Yes | Reported | No (NA) | NA | 6 |
| Chow (2022) | Yes | Yes | Secure-Record | Yes | Yes | Reported | Yes | Yes | 8 |
| Formiga (2021) | Yes | Yes | Secure-Record | Yes | Yes | Reported | Yes | Not reported | 7 |
| Aghajani (2021) | Yes | Yes | Secure-Record | Yes | Yes | Reporte | Yes | Yes | 8 |
| Vahedian-Azimi (2021) | Yes | Yes | Secure-Record | Yes | Yes | Reported | No (NA) | Yes | 7 |

**Table 2. Quality of included studies as shown by the Newcastle-Ottawa Scale**

NA: Not applicable

After using the Newcastle-Ottawa Scale we found that all the included studies truly had aspirin as the exposure and were selected using appropriate definition of exposure in hospitalized COVID-19 patients (17). The non-exposed were non-aspirin exposed group with COVID-19 hospitalization. The study also controlled for confounders and seven of the ten studies had follow up long enough for the outcome to occur. All the included studies had score more than or equal to six which proves the study were of high quality.

**Figure 4: Risk of bias summary for the included randomized controlled trial.**

**Figure 4** depicts the assessment of the risk of bias in the Randomized Controlled Trial included in our study using the Cochrane risk of bias tool (18). As mentioned in the methods section above, the summary is color coded with green representing a low risk of bias, yellow- unclear risk of bias (If insufficient detail is reported of what happened in the trial, the judgment will usually be an unclear risk of bias), and red- high risk of bias. Selection bias has two domains- Random sequence generation and Allocation concealment. These domains in the selection bias describe the methods implemented for randomization to produce comparable groups for the study and concealment of intervention allocation in the comparison groups. Performance bias describes the measures used for blinding the trial participants and researchers regarding the intervention participants received. Detection bias describes measures adopted to blind the outcomes assessment. Attrition bias describes the completeness of the outcome data including the reasons for attrition and exclusions from the analysis. Reporting bias states selective outcome reporting in the trial, if any. Other biases illustrate any other important concerns that are not specified in other domains of the risk of bias tool (18).

## 4. Discussion:

Our study investigated the role of aspirin on all-cause mortality in COVID-19 hospitalizations, including a subgroup of the role of aspirin in in-hospital mortality of the recently published RECOVERY trial (3) and a large observational study by Chow et al.(11) Our results show that hospitalized patients for COVID-19 taking aspirin had lower all-cause mortality compared to those not taking aspirin. Notably, the RECOVERY trial did not demonstrate improvement in 28-day mortality with aspirin use in hospitalized

COVID-19 patients; however, the proportion of patients discharged alive within 28 days was higher in patients who received aspirin (75% vs. 74%, rate ratio: 1.06, 95% CI, 1.02-1.10, p=0.006) (3). This finding is similar to our study where aspirin improved in-hospital mortality in patients hospitalized with COVID-19.

COVID-19 contributes to a prothrombotic and hypercoagulable state (8-9). Increased production of interleukins (IL-6, IL-10) and coagulopathy leads to high fatality rates in hospitalized COVID-19 patients. (28-29) Studies have shown systemic anticoagulation's benefits in reducing mortality in mechanically ventilated patients. (30-31). Aspirin has anti-inflammatory, antiplatelet, and antiviral effects which have shown in both in-vitro and experimental models to reduce replication, propagation, and infectivity of many RNA viruses such as MERS-CoV and CoV-229 E (4, 9-12). Hence, aspirin was studied as one of the therapeutic options in patients with COVID-19.

While previous meta-analyses have been conducted (15, 32-35), none have been restricted to hospitalized COVID-19 patients and the outcome of in-hospital mortality. Namely, Osborne et al. conducted a large observational study showing improved mortality in COVID-19 with aspirin; however, the patients were enrolled in the Veterans Affairs health system and were not limited to hospitalized COVID-19 patients (34).

Our study's results on all-cause mortality in hospitalized COVID-19 patients align with the previous meta-analyses (32-35); however, our study is unique in reporting both in-hospital mortality and overall mortality among these patients. Moreover, our study includes updated data with the inclusion of a recent large observational study by Chow et al.(11) In an earlier study by Chow et al. of 412 patients hospitalized with COVID-19, low-dose aspirin use was associated with decreased need for mechanical ventilation and intensive care unit admission as well as in-hospital mortality after multivariable adjustment.(4) Similarly, a single-center study by Liu et al. showed that low-dose aspirin prevents embolic events in patients infected with COVID-19 while decreasing mortality (12). The studies by Yuan et al.(14), Alamdari et al.(26), Formiga et al.(24), and Vahedian-Azimi et al.(23) were the observational studies in our meta-analysis that did not show improved all-cause mortality. Alamdari et al. conducted a retrospective cross-sectional study with a higher risk of bias and lack of adjustment for potential confounders, which may have contributed to different results (26). Yuan et al. investigated pre-hospitalization use of low-dose aspirin in COVID-19 patients with coronary artery disease (14). The group with pre-hospitalization use of aspirin who continued aspirin in the hospital may have thus been sicker than the non-aspirin cohort leading to higher in-hospital mortality. Moreover, the smaller sample size, differences in comorbidities, and non-generalizable populations like Spanish, Italian, and Iranian populations could have led to different outcomes. (23-28)

Our study has several limitations. We had only one RCT; the remaining ten were observational studies, with one cross-sectional study prone to unmeasured confounders. In most of the included studies, the severity of COVID-19 infection is not mentioned; however, all patients required hospitalization, meeting the criteria for moderate to severe COVID-19. We also didn't report other outcomes like bleeding, which may occur with aspirin. With meta-analyses, there is always a concern for reporting biases, such as selective outcome reporting.

Nonetheless, included studies used propensity scores to reduce confounding and selection bias, and we used adjusted hazard ratios for accurate results from included cohort studies. There was significant heterogeneity among the studies. Furthermore, patients taking aspirin in observational studies are generally more likely to have cardiovascular disease, which may place them at higher risk for mortality, which may have reduced the mortality benefit seen in our study. Finally, the included studies were single-center studies or registry data from China, Spain, Iran, Italy, or the United States, except for the RECOVERY trial (3). This limitation can affect the generalizability of the study to other ethnic groups.

## 5. Conclusion:

Our study shows that aspirin decreases in-hospital mortality in patients hospitalized with COVID-19. Further studies are needed to assess which COVID-19 patient populations benefit most, such as patients on aspirin for primary vs. secondary prevention of atherosclerotic disease. In addition, significant bleeding also needs to be considered when assessing the risk-benefit of aspirin use.

**6.** Acknowledgment**:** None

156x173mm (150 x 150 DPI)

| Study or Subgroup | log[Risk Ratio] | SE | Weight | Risk Ratio IV, Random, 95% CI |
|---|---|---|---|---|
| **1.1.1 In-hospital mortality** | | | | |
| Aghajani et al. 2021 | −0.2877 | 0.14 | 11.5% | 0.75 [0.57, 0.99] |
| Alamdari et al. 2020 | 0.207 | 0.2753 | 4.5% | 1.23 [0.72, 2.11] |
| Chow et al. 2020 | −0.6349 | 0.2702 | 4.6% | 0.53 [0.31, 0.90] |
| Chow et al. 2022 | −0.145 | 0.0399 | 23.1% | 0.87 [0.80, 0.94] |
| Formiga et al. 2021 | 0.0488 | 0.0639 | 20.3% | 1.05 [0.93, 1.19] |
| Meizlish et al. 2021 | −0.6539 | 0.2261 | 6.1% | 0.52 [0.33, 0.81] |
| Vahedian-Azimi et al. 2021 | −0.2744 | 0.4728 | 1.7% | 0.76 [0.30, 1.92] |
| Yuan et al. 2020 | −0.0408 | 0.3292 | 3.3% | 0.96 [0.50, 1.83] |
| **Subtotal (95% CI)** | | | **75.1%** | **0.84 [0.71, 0.99]** |
| Heterogeneity: $Tau^2 = 0.02$; $Chi^2 = 19.57$, df = 7 (P = 0.007); $I^2 = 64\%$ | | | | |
| Test for overall effect: Z = 2.10 (P = 0.04) | | | | |
| **1.1.2 Mortality** | | | | |
| Liu et al. 2021 | −1.6607 | 0.7005 | 0.8% | 0.19 [0.05, 0.75] |
| RECOVERY Collaboration 2021 | −0.0408 | 0.0408 | 23.0% | 0.96 [0.89, 1.04] |
| Viecca et al. 2020 | −0.5978 | 0.6134 | 1.0% | 0.55 [0.17, 1.83] |
| **Subtotal (95% CI)** | | | **24.9%** | **0.56 [0.22, 1.41]** |
| Heterogeneity: $Tau^2 = 0.44$; $Chi^2 = 6.13$, df = 2 (P = 0.05); $I^2 = 67\%$ | | | | |
| Test for overall effect: Z = 1.22 (P = 0.22) | | | | |
| **Total (95% CI)** | | | **100.0%** | **0.86 [0.76, 0.97]** |
| Heterogeneity: $Tau^2 = 0.02$; $Chi^2 = 27.40$, df = 10 (P = 0.002); $I^2 = 64\%$ | | | | |
| Test for overall effect: Z = 2.37 (P = 0.02) | | | | |
| Test for subgroup differences: $Chi^2 = 0.69$, df = 1 (P = 0.41), $I^2 = 0\%$ | | | | |

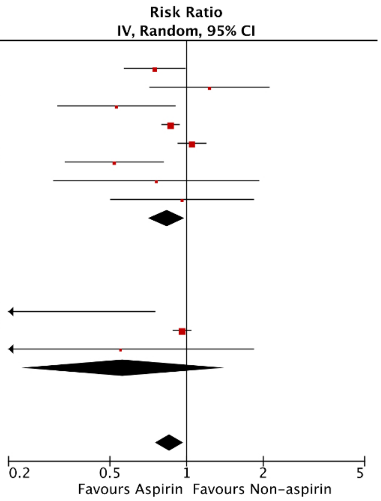

Figure 2: Forest plot of the effect of aspirin use on overall and in-hospital mortality in adults hospitalized with COVID-19.
CI: Confidence Interval SE: Standard Error

226x114mm (150 x 150 DPI)

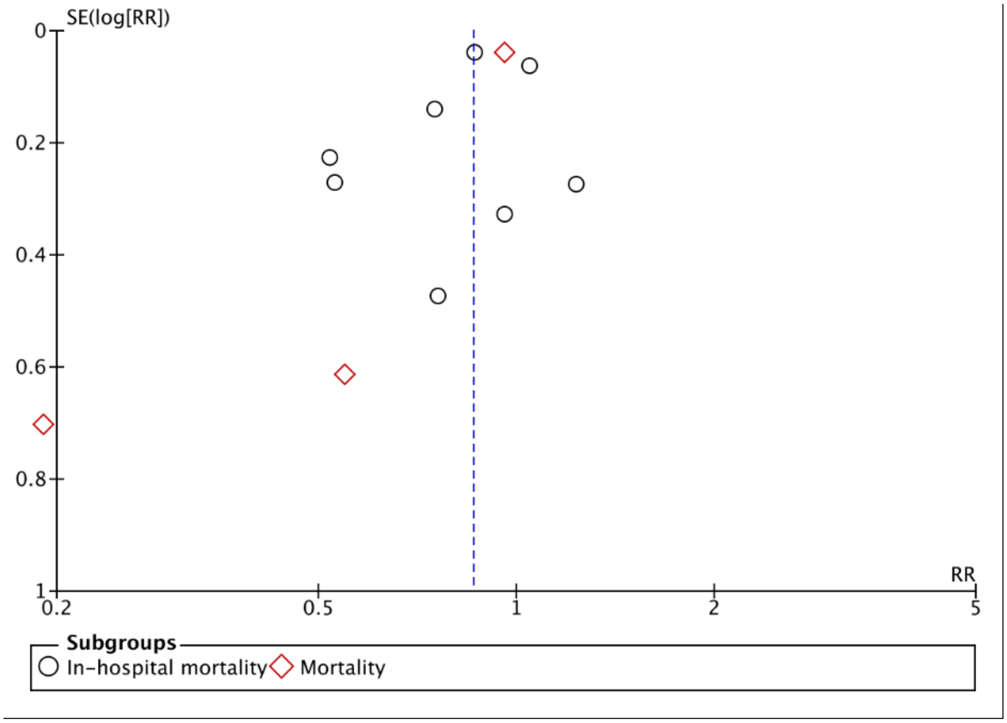

Figure 3: Funnel plot for the assessment of publication bias of the included studies. The blue dotted line represents the odds ratio of all-cause mortality. SE: Standard of the Error log: logarithmic RR: Relative Risk

159x113mm (150 x 150 DPI)

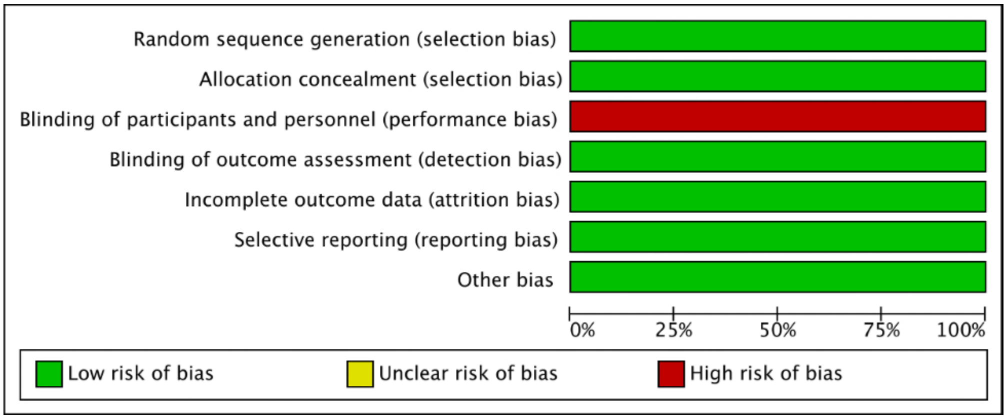

Figure 4: Risk of bias summary for the included randomized controlled trial.

164x67mm (150 x 150 DPI)

## Supplementary Material

bpac027_Supplementary_Data

